# A Multi-Omics Study of Human Testis and Epididymis

**DOI:** 10.3390/molecules26113345

**Published:** 2021-06-02

**Authors:** Weimin Zheng, Yang Zhang, Chuanyu Sun, Shengyang Ge, Yifan Tan, Huali Shen, Pengyuan Yang

**Affiliations:** 1 Department of Chemistry, Fudan University, Shanghai 200433, China; wmzheng6@163.com; 2 Department of Systems Biology for Medicine and Institutes of Biomedical Sciences, Shanghai Medical College, Fudan University, Shanghai 200032, China; zhangyang@fudan.edu.cn; 3 Department of Urology, Huashan Hospital, Fudan University, Shanghai 200040, China; zhugexianglong@163.com (C.S.); 12301010002@fudan.edu.cn (S.G.); tyf930501@163.com (Y.T.)

**Keywords:** proteomics, transcriptome, missing protein, beta-defensin, MRM technique

## Abstract

The human testis and epididymis play critical roles in male fertility, including the spermatogenesis process, sperm storage, and maturation. However, the unique functions of the two organs had not been systematically studied. Herein, we provide a systematic and comprehensive multi-omics study between testis and epididymis. RNA-Seq profiling detected and quantified 19,653 in the testis and 18,407 in the epididymis. Proteomic profiling resulted in the identification of a total of 11,024 and 10,386 proteins in the testis and epididymis, respectively, including 110 proteins that previously have been classified as MPs (missing proteins). Furthermore, Five MPs expressed in testis were validated by the MRM method. Subsequently, multi-omcis between testis and epididymis were performed, including biological functions and pathways of DEGs (Differentially Expressed Genes) in each group, revealing that those differences were related to spermatogenesis, male gamete generation, as well as reproduction. In conclusion, this study can help us find the expression regularity of missing protein and help related scientists understand the physiological functions of testis and epididymis more deeply.

## 1. Introduction

The mammalian testis and epididymis are two main organs of the male reproductive system [1]. The main function of the testis is to produce spermatozoa for reproduction [2]. Once released from the testis, the spermatozoa are transferred to the epididymis. During passing through the epididymis, spermatozoa interact with the secreted epididymal proteins that promote maturation, including the acquisition of motility and fertilization competence [3]. Yet, the testis and epididymis have evolved a series of structural and functional features that are far beyond the scope of our knowledge.

Focused on the specific tissue, decades of research revealed that the quantities of proteins vary from greatly across different tissues [4,5]. The testis has high gene expression levels, and the genome-wide analysis of testis showed lists of genes expressed in a testis-enriched pattern [6,7]. Several studies have tried to identify proteins and protein-coding genes expressed in testis from RNA-Seq and mass spectrometry; for example, Liu et al. had identified 7346 proteins in human testis by using an advanced proteomics platform [8]. The outbreak of COVID-19 caused by SARS-CoV-2 has infected millions of individuals around the world. Current studies confirm that SARS-CoV-2 can affect the male reproductive system. Nie et al. revealed the reduction of Leyding cells, impairment of spermatogenesis and sperm motility in the testis by using the proteomic analysis [9]. In addition, the high expression of the receptor angiotensin-convertng enzyme 2 (ACE2) on the surface of spermatogonia and supporting cells in the testis can lead to testicular spermatogenesis dysfunction and reduced sperm count [10].

The number of functional human protein-coding genes in the reference genome is about 20,000 [11,12]. Based on the nextprot database (version 2020.1), there are still about 9.3% of the proteins in the human proteome that have been confidently predicted but have not been detected or confirmed accurately by MS, Ab-based methods, or other methods. The Chromosome-Centric Human Proteome Project (C-HPP) is organized in the study of the human proteome on a chromosome-centric basis, and the target mission is focusing on the discovery and characterization of these so-called missing proteins (MPs) [13,14,15,16]. For example, the beta-defensin family, which is composed of 34 members (MV almost in 10,000 Da), is highly absent in previous proteomic experiments but plays critical roles in innate immunity [17,18]. Therefore, it is challenging but meaningful work to complete the missing puzzles by confirming the missing proteins and revealing their physiological functions.

Due to the high sensitivity, broad dynamic range, and reproducibility in quantifying proteins, multiple reaction monitoring (MRM) was accepted by C-HPP as one of the effective methods to identify and validate the MPs [19,20]. Recently, multi-omics analysis of transcriptomic and proteomic data has become a new promising method for noting the gene function and finding missing proteins, through revealing unknown biological regularities and underlying the discipline during gene translation [21].

While there are unique functions of the two organs, to the best of our knowledge, the proteomic analysis of epididymis has not been explored, and there also had not integrative analysis of transcriptomes and proteomes across testis and epididymis. Therefore, the purpose of this study was to generate a resource of profiling data at the protein and mRNA level by multi-omics analysis to facilitate the study of proteins involved in spermatogenesis and sperm mutation and provide a systematic and comprehensive proteome and transcriptome study between testis and epididymis. To this end, the in-depth proteomic analysis provided us a new idea to identify MPs.

## 2. Results and Discussions

### 2.1. Overall Workflow for Analyzing Testis and Epididymis Samples

**Figure 1:**
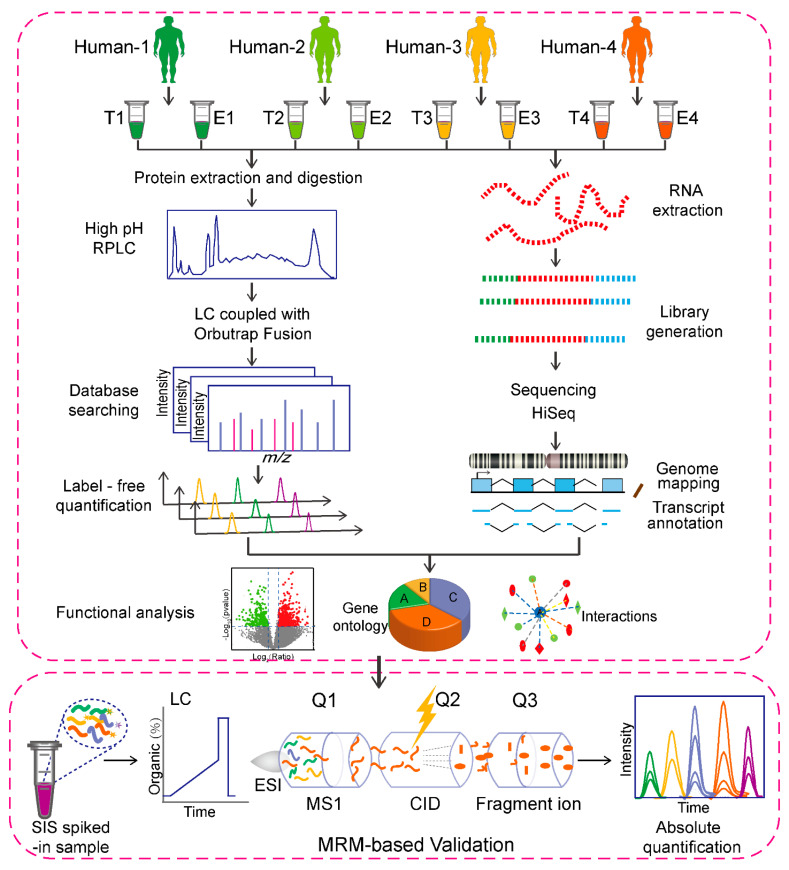
Experimental workflow for the proteomics and transcriptional analysis of testis and epididymis samples, and further validation of selected proteins by utilizing the MRM technique.

Although C-HPP groups have intensively studied human testis, the epididymis, a region of the male productive tract that contributes to sperm maturation and survival, has not been fully studied. Our overall workflow for the detection and validation of missing proteins is illustrated in Figure 1. Testis and epididymis were both chosen as the tissue samples for studying the deep proteome profile as well as mining the missing proteins. After extracting the proteins from the testis and epididymis via the optimized Lys-C/Trypsin digestion, we utilized the C_18_-based HP-RP prefractionation coupling with LC-MS/MS to increase the protein coverage, followed by the operation of database searching and label-free quantitation of proteins. Overall, we achieved the efficient identification of proteins from the testis and epididymis by using this strategy. The optimized process successfully provided a high protein coverage within the individual male productive tract. In the transcriptome analysis, the high-quality RNA-SEQ data were also obtained from the workflow, as shown in Figure 1. We further performed a comparative analysis between the proteomic and transcriptomic data of testis and epididymis by using several informatics tools, and the details were described in the following sections. Furthermore, the strategy mentioned above was also adopted to mine the missing proteins, and we finally confirmed the existence of 110 missing proteins, some of which belonged to the beta-defensin protein family. Then, the MRM technique was used to validate these missing proteins.

### 2.2. Proteomic Data Analysis of Testis and Epididymis Tissues

**Figure 2:**
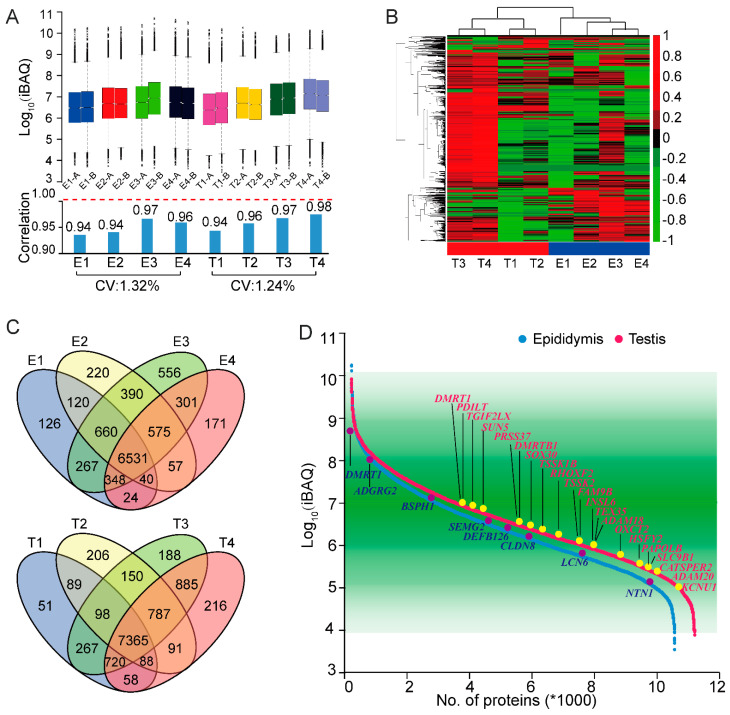
The proteomics analysis of the testis and epididymis. (**A**) Top, boxplot analysis of the sample is illustrated. The sample is presented on the horizontal axis and the normalized amounts of expression are shown on the vertical axis. Bottom, the coefficient of variation of proteins in the two technical repetitions and the same tissues. (**B**) Heat map showing a pairwise correlation between proteins across all individuals, with positive correlation in red and negative correlation in green. (**C**) Venn diagram of proteins identified in testis and epididymis. (**D**) Distribution of protein quantitative value. Proteins are ranked by iBAQ value and the dynamic range of protein level spanned over six orders of magnitude in testis and epididymis. The * means the number multiply by 1000. Several proteins of interest are highlighted in red and blue; a complete list is provided in the dataset.

We generated a high-quality and high-resolution mass spectrometry dataset from the testis and epididymis. Testis and epididymis tissues from four peoples were involved for proteomic and transcriptomic analysis, and each sample was analyzed twice. For the analyses of the testis, 8736, 9661, 10,460, and 10,210 proteins were identified for each sample, whereas 8116, 8593, 9628, and 8047 were observed from each of the epididymis tissue (Table S1 in the Supplementary Materials). Taken together, a total of 11,024 and 10,386 proteins were identified from the testis and epididymis, respectively. The Venn diagram in Figure 2C shows the comparison of proteins identified within the testis and epididymis. The Venn diagram shows an overlap of 66.8% (7365 out of 11,024) and 62.9% (6531 out of 10,386) across each sample in epididymis and testis, respectively.

To assess the data quality and reliability prior to replicate experiments, a boxplot analysis of samples is shown in Figure 2A. The overall protein expression profiles of testis and epididymis were comparable as a median center distribution. The correlation coefficients between each technical repetition were above 0.99, the variation coefficient of testicles tissue and epididymis tissue (coefficient of variation, CV) was 1.32% and 1.24%, respectively. The above results showed that our experiment has good repeatability. Then, we investigated the global protein expression profiles by using the hierarchical clustering method, which was based on the correlation among eight biological replicates (Figure 2B).

A spectral counting-based label-free approach was used for quantitative by maxquant (version 19). The abundance of identified proteins spanned over six orders of magnitude (Figure 2D). The gradation of green color was used to visualize the number of proteins in a certain region. The 21 testis-specific proteins and eight epididymis-specific proteins were annotated in the figure. These proteins were mainly involved in the transcription regulation and protein transportation with the generation of sperm, such as transcription protein DMRT1, PDILT, TGIF2LX, SUN5, PRSS37, DMRTBI, SOX30, TSSK1B, RHOXF2, TSSK2, etc. Gene Ontology (GO) (version 6.8) was used to analyze each level of proteins, and we found that high-abundance proteins in the testes were mainly involved in the self-assembly of protein complexes, positioning process of cell protein, and adhesion among cells. High-abundance proteins in the epididymis also participated in the redox process and fatty acid metabolism process. For the medium abundance proteins, they were mainly mitochondrial proteins in the testes, which are involved in energy metabolism processes, protein positioning, and transportation, synthesis, and assembly of in-cell compounds. Meanwhile, the proteins in the epididymis are mainly located in the mitochondria and extracellular matrix. They were responsible for the transport and location of proteins, and synthesis and metabolism of amino compounds.

### 2.3. Relative Quantitative Comparison of Proteome among Testis and Epididymis

**Figure 3:**
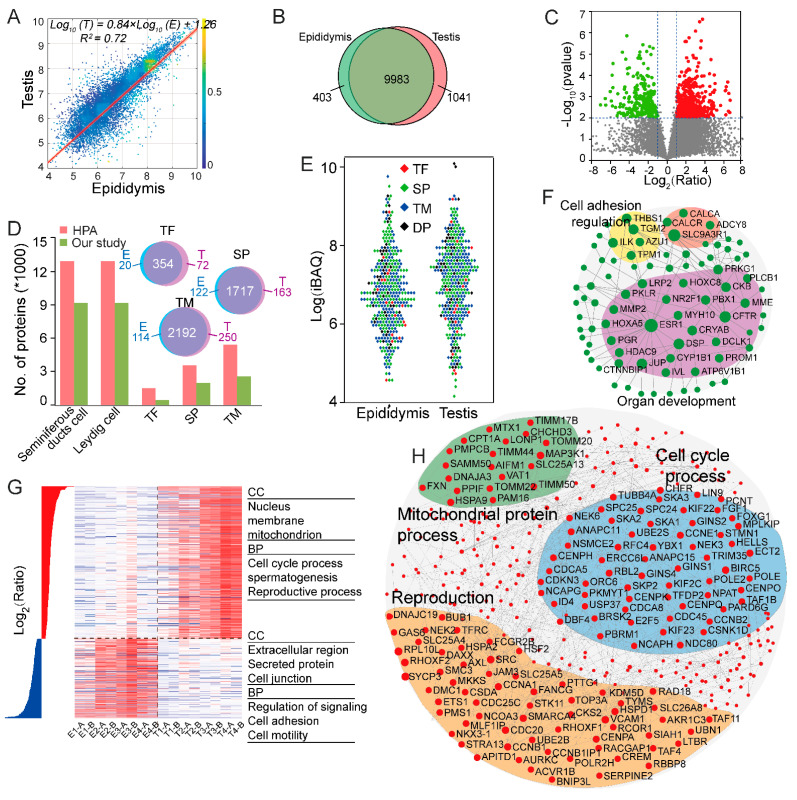
Comparative analysis of testis and epididymis protein abundance. (**A**) Pairwise comparisons of proteomes across testis and epididymis. (**B**) The Venn diagram represents the overlap of the testis and epididymis. (**C**) Volcano plots illustrate pairwise differential expression changes (using log_2_ median-centered values) between testis and epididymis. (**D**) Our dataset (protein set) was compared to the protein list from HPA using the histogram. Venn diagram represents the overlap of three kinds of proteins (TF, SP, and TM) across testis and epididymis. (**E**) The warm bees plot of three types of proteins in testis and epididymis. (**F**) Differentially expressed genes were analyzed by NetworkAnalyst, resulting in the biggest network in the epididymis (**G**) Hierarchical clustering of differentially expressed proteins in four types of two tissues, and GO annotation of these two parts of proteins. (**H**) Network plot of high expressed proteins in the testis and the enriched genes of the same biological process were displayed in the same colors.

As shown in Figure 2A, the correlation coefficient between the proteomes of testis and epididymis was 0.72, reflecting conserved protein expression. The Venn diagram showed 9983 commonly identified proteins in both tissues, accounting for 87.4% of the total identified proteins. In addition, 1041 proteins were only identified in testis and 403 proteins were unique in the epididymis (Figure 3B). For relative quantitative analysis, with a criterion of the *p*-value (<0.01) and fold change greater than 2500 proteins were upregulated in the epididymis, while 260 proteins were downregulated compared to the testis (Table S2 in the Supplementary Materials). A volcano plot of all quantified proteins is shown in Figure 3C, displaying the relationship between statistical significance (−log p value) and log_2_ ratio of each protein. The green dots and red dots represented that the proteins had statistically significant differential expression.

GO annotation tools were used to analyze the differential proteins. The cellular component (CC) terms of testis-enriched proteins were nucleus, membrane, and mitochondrion. Whereas, the dominant CC categories were identified as extracellular region, secreted protein, and cell junction in the epididymis. As might be expected, the enriched GO biological process (BP) categories of the testis-enriched proteins were cell cycle process, spermatogenesis, and reproductive process. Meanwhile, the specific terms were regulation of signaling, cell adhesion, and cell motility in the epididymis (Figure 3G).

To assess whether our data were reliable and had deep coverage of proteins, we compared the proteomic results with the the HPA database. The results showed that the sample for analysis here was relevant with seminiferous ducts cell or Leyding cell in the HPA database. In our study, we confirmed 9226 proteins and shared 71.1% of proteins with seminiferous duct cells in the HPA database (Figure 3D). Our work identified a large number of low-abundant transcription factors, secret proteins, and transmembrane proteins with crucial biological functions. These proteins had a high overlap in the two tissues and the full lists of the proteins that were provided in Table S3 in the Supplementary Materials. The Scatter diagram was used to visualize the relative abundance of these three kinds of proteins. As shown in Figure 3E, the median expression of these three types of proteins in the testis is higher than in epididymis, which could be attributed to the fact that the cells of testis need energy and materials transport in the spermatogenesis process.

To explore the functional characteristics and network of differentially expressed proteins in testis and epididymis, the networks were constructed and visualized by NetworkAnalyst. The top module in the samples was selected, and the KEGG pathway enrichment analysis revealed the genes involved in a similar function and marked as the same color in the module. As showed in Figure 3F, a total of 83 proteins were in the top module of the epididymis. The significant signaling pathways were enriched in organ development (30 proteins), cell adhesion regulation, and G-protein coupled receptor (GPER). It is reported that GPER has been found in the male reproductive of many mammalian species and is involved of several essential processes during sperm development and maturation [22], whereas the top module had 342 proteins in testis. These genes were mainly enriched in the cell cycle process, mitochondrial protein, and reproduction, based on the enrichment analyses, as shown in Figure 3H. The meiotic cell cycle is specialized in testis, and the meiotic is a complex process with high energy demands. Mitochondria are dynamic throughout the cell cycle, which involved the dramatic upregulation of thousands of proteins in mitochondria as well as the cell cycle process [23].

### 2.4. Multi-Omics Data Integration in Testis and Epididymis

**Figure 4:**
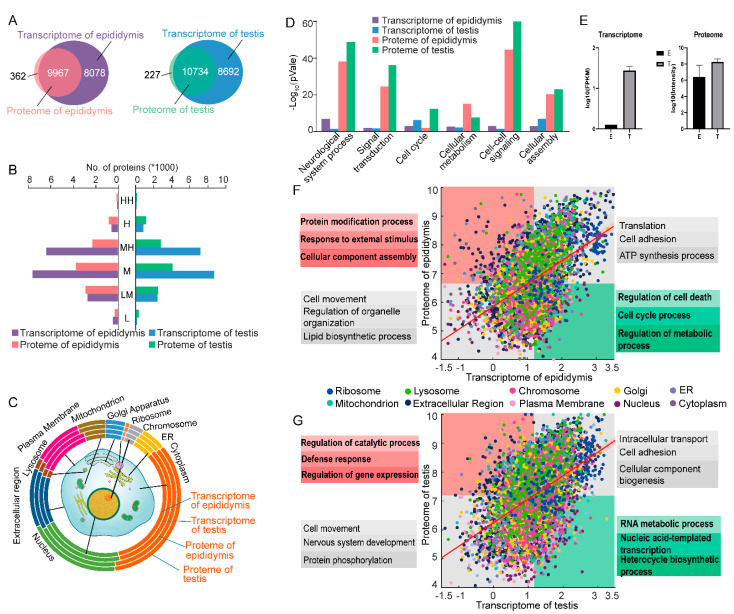
Integration of transcript and proteomic analysis. (**A**) Venn diagram of proteome and transcriptome; (**B**) Distribution of gene expression at the different levels; (**C**) Cellular component analysis of proteome and transcriptome; (**D**) GO annotation of the aggregation of transcription and proteomics of both tissues; (**E**) Analysis of ACE2 expression in testis and epididymis transcriptome and proteome profile; (**F**,**G**) Analysis of the correlation between proteomics and transcriptional data.

By systematically analyzing high-throughput RNA-SEQ data, we generated 13,000 protein-coding genes with a cut-off of 1 fragment per kilobase million (FPKM). As shown in Figure 4A, 98% of proteins had transcripts supporting. To describe the expression of transcripts and proteins, we divided them into six concentration classes, Low (L), Low Middle (LM), Middle (M), High (MH), High (H), and High High (HH). As shown in Figure 4B, 85% of the total proteins were in the LM, M, and MH levels, whereas in the transcriptome, the percent was 90. Furthermore, the subcellular distribution of the testis and epididymis is shown in Figure 4C. The proteome of the testis, proteome of the epididymis, the transcriptome of the testis, and transcriptome of the epididymis are presented in the circle from outside to the inside, respectively.

The top six enriched biological processes are graphed in Figure 4D. The genes in proteomic datas were more intensively enriched in these processes than in the transcriptome, including neurological processes, signal transduction, intercellular cell signaling, and cell differentiation. The two processes, signal transduction and intercellular signaling process, had higher expressed in the testis than in the epididymis. Proteins related to the cell cycle and cell differentiation process were also highly expressed in the testis. The key function of these involved proteins was for the production of sperm cells.

By integrating the mass spectrometry-based proteomics and RNA-Seq technology, it would enable a comprehensive analysis of factors explaining the experimentally observed differences between mRNA and protein expression. We examined pairwise relationships between RNA and protein by integrating transcriptomic and proteomic data types. As shown in Figure 4F,G, the mRNA–protein relative analysis was explored by using a scatter plot in the testis and epididymis. The x-axis represented the transcription, and the y-axis was for proteomics. Four regions were divided by using the median data. The red area represented a higher transcription efficiency rate, while the green region represented a low transcription efficiency rate. The gray areas represented proteins–transcript pairs with consistent expression trends. The color of the plot represented the cellular composition of different genes. There were 466 pairs in the high-efficiency zone for the epididymis; the cellular component analysis showed that 182 genes were mainly in the membrane-bound vesicle, and 189 were the extracellular regions. Biological process analysis indicated that the genes primarily participated in the protein modification process, response to external stimulus, cellular component assembly. Meanwhile, the genes in the green region were involved in the regulation of the cell death, cell cycle process, and regulation of the metabolic process. These results showed that the maturation process of spermatozoa required the interaction with epididymal secretory proteins and post-translational modification when the spermatozoa pass after the testis [24]. As shown in Figure 4G, 659 genes were in the red region in the testis, 261 genes were expressed in the extracellular region and mainly participated in the regulation of the catalytic process, defense response, and regulation of gene expression. The genes in the green region were mainly involved in the RNA metabolic process, nucleic acid-templated transcription, heterocycle biosynthetic process in the testis. The results indicated that gene expression produced unique and characteristic groups of proteins that provided greater insight into activities, biological of testicular cell types during the progression of spermatogenesis in testis [25].

ACE2 is a receptor of the SARS-CoV-2 virus and played a critical role in the viral entry into the cells during the virus infection process [26]. Recently, studies have shown that ACE2 expression distributions were organ-specific, mainly in the kidney, testis, and breast, cardiovascular and gastrointestinal systems [27]. In our study, ACE2 was presented in testis both in transcription and proteome, and ACE2 had a high expression in the transcript but not in the protein level in the epididymis (Figure 4E). Taken together, this study may provide evidence that the testis is one of the main target tissues of SARS-CoV-2 virus. It may have an impact on male fertility, especially in the spermatogenesis process.

### 2.5. Identified Missing Proteins in Testis and Epididymis Samples

**Figure 5:**
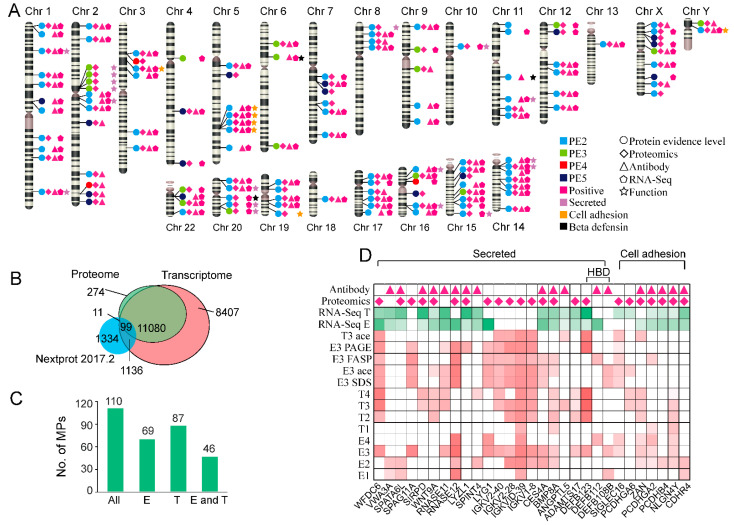
Proteomics analysis of missing proteins. (**A**) The distribution of the identified missing proteins on chromosomes; (**B**) Venn diagram of testis and epididymis with the database; (**C**) The number of missing proteins in both tissues; (**D**) Functional analysis of genes identified by transcription groups to missing proteins and their comparison with data; and identification results and functional analysis of missing proteins in each sample. The depth of the red color represents the protein expression, and the green squares in the figure represent the amount of expression of transcription groups in both tissues.

According to the neXtProt database (2018.1.20), there are 1924 MPs that the protein level is at protein evidence 2-4 (PE 2-4). In our study, RNA-Seq data showed that 1238 genes correspond to missing genes in the testis, and 1042 genes were in the epididymis (Figure S1). The biological function of these genes was mainly enriched in the membrane, DNA binding, zinc finger, etc. This result confirmed that the male productive organs had expressed more specific proteins than normal tissues. It was promising that a significant number of missing proteins would be identified in the testis and epididymis proteomics. A total of 110 missing proteins were identified with two proteotypic peptides and the length of ≥9 amino acids, and the list is summarized in Table S4 in the Supplementary Materials. Among them, 69 and 87 missing proteins were respectively identified in the epididymis and testis, 46 of which were simultaneously identified in both tissues (Figure 5B). All these candidates were mapped on the 24 chromosomes (Figure 5A). The status evidence for the existence of these proteins was added to the chromosome with a small circle. The gene evidence of these MPs was presented, including the proteomics, antibody, antibody, and RNA-SEQ evidence.

Furthermore, GO analysis was performed to reveal the functional annotations of partially missing proteins. Twenty-three proteins belonged to secreted proteins, including three beta-defensins and seven proteins responsible for cell adhesion. The expression level of mRNA and protein in the different experiments was visualized in Figure 5D. As we can see, most proteins were detected at the transcriptional level. A large number of proteins were presented in two or more individuals. The chromosome Y is essential for male fertility. In our study, 14 Y-linked genes were detected at the transcriptional level and 12 Y-linked genes were at the protein level. Of them, the proteins Neuroligin-4 and TSPY (testis-specific protein, Y-encoded) were missing. TSPY is a testis-specific protein and may play a pivotal role in the etiology of testis cancers [28].

### 2.6. Validation of Beta-Defensin Using MRM Technique

The beta-defensin family belong to antimicrobial peptides. Some beta-defensins are expressed in the reproductive tract and may play a dual role in fertility, host defense, and sperm maturation. Their absence can lead to a reduction in sperm’s ability to swim [29,30]. However, the detailed mechanism in these processes is not particularly clear [31]. Most of the beta-defensins are lower than 10 kDa, and there are only three or five peptides suitable for detection by mass spectrometry. The identification of beta-defensins is critical for studying the functions. After manual inspection of the spectra in these two tissues, we confirmed seven beta-defensins, including DEFB106, DEFB118, DEFB119, DEFB121, DEFB126, DEFB129, and DEEFB132, which were now considered as known proteins. Five beta-defensins that belonged to MPs were identified in our study. Among them, protein DEFB 108, DEFB112, and DEFB123 had two proteotypic peptides of ≥9 amino acids, whereas DETB104 and DEFB105 were respectively identified by one unique peptide.

**Figure 6:**
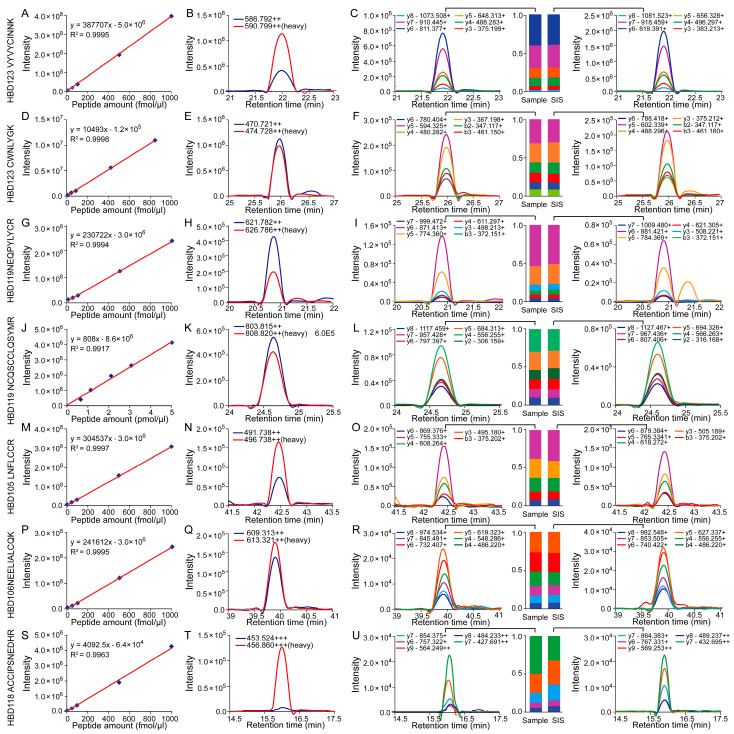
The validation of beta-defensins based on the MRM technique. The linear of the SIS peptides were displayed in the (**A**,**D**,**G**,**J**,**M**,**P**,**S**). The TIC diagrams of the SIS peptides and the endogenous peptides segments were displayed in the (**B**,**E**,**H**,**K**,**N**,**Q**,**T**). The endogenous and SIS peptides were calculated and compared according to the MS2 traces, and showed in (**C**,**F**,**I**,**L**,**O**,**R**,**U**).

To confirm the missing proteins in testis, we synthesized seven SIS (stable isotope-labeled peptides) corresponding to five beta-defensins for the validation with MRM experiments. On the basis of the SIS peptides and the natural peptides being co-eluted by the liquid chromatography [32], the MS/MS fragment patterns of the peptides in samples were exactly matched with the SIS peptides (Figure 6). To determine the absolute concentration of these proteins in the tissues, we added four to five concentration gradients to the sample, and each concentration was repeated three times. The technical reproducibility was pretty high, and the R^2^ between technical replicates ranged from 0.9917 to 0.9997 (Figure 6). According to the MRM results, we quantified five beta-defensin proteins in the tissues, with quantitative results shown in Table 1. As we can see, the HBD123 was significantly highly expressed in testis, with a median concentration at 0.9 pmol/uL in the testis sample.

## 3. Materials and Methods

### 3.1. Tissue Preparation and Protein Extraction, Digestion, and Fractionation

Clinical human samples were obtained from HuanShan Hospital, in accordance with approved human subject guidelines authorized by medical ethics and the human clinical trial committee at the hospital. In this study, four pairs of testis and epididymis from four individual patients were used for analysis. Following surgery, the adjacent normal tissues were collected in separate tubes stored at −80 °C until use. After frozen tissue was cut into small pieces and washed by PBS to remove blood, the precleaned tissues were ground to powder with nitrogen and sonicated in lysis buff (buffer A: 7 M urea, 2M thiourea, 2% (*w*/*v*) CHAPS; with protease inhibitor cocktail (50:1, sample/protease inhibitor, *v*/*v*) on ice, respectively. Then, they were centrifuged at 140,000 g for 30 min to obtain the supernatant. In-solution digestion: to enhance the digestion performance, we applied the Lys-C/Trypsin combined enzymatic method in protein digestion. After proteins were reduced with 10 mM DTT and alkylated with 20 mM IAA, endoproteinase Lys-C was added into protein at the mass ratio of 1:50 (*w*:*w*) enzyme: protein ratio and incubated for 3 h at 37 °C. Next, the samples were diluted with 50 mM ammonium bicarbonate until the concentration of urea was below 1 M and trypsin was added to the sample at the mass ratio of 1:30 (*w*:*w*) enzyme: protein for 12 h. The digested peptides were desalted by means of Sep-Pak C 18 columns (Waters Corporation, Milford, MA, USA) according to the manufacturer’s instructions and then resuspended in 2% acetonitrile/0.1% formic acid. Then, the peptides were fractionated separately by using high pH reversed-phase liquid chromatography on a UPLC system (Waters Corporation, Milford, MA, USA).

### 3.2. LC-MS/MS Analysis of Proteins and Data Analysis

LC-ESI-MS/MS analyses were performed using a Nano ACQUITY system (Waters Corporation, Milford, MA, USA) coupled to an Orbitrap Fusion mass spectrometer (Thermo Fisher Scientific, Waltham, MA, USA). A two-column setting was adopted for all analyses. Samples were firstly loaded onto the C18 Nano Trap Column and then analyzed on a C18 column (Thermo Fisher Scientific, 150 mm × 75 µm, 3 µm). The mobile phases consisted of Solution A (0.1% formic acid) and Solution B (0.1% formic acid in ACN). The derivatized peptides were eluted using the following gradients: 5–28% B at a flow rate of 200 nL/min in 90 min, 28–35% B in 10 min, 90% B for 20 min. Survey scans of peptide precursors from 350 to 1,600 *m*/*z* were performed at *m*/*z* 400 resolution with a 10^5^ ion count target. The 15 most abundant ions in each MS scan were automatically selected and fragmented in HCD mode to achieve high mass accuracy in MS/MS spectra. For MS/MS analysis, the isolation window was set as 2.0 Da, normalized collision energy as 30.0, activation time as 0.1 ms, and the starting mass as 100.0Da.

The raw data were processed by MaxQuant version 1.5.3.8 software (Martinsried, Bavaria, Germany), using the Andromeda search engine against the human database (Version May 2017) selecting trypsin digestion. The carbamidomethyl of cysteine was specified as a fixed modification. The oxidation of methionine and acetyl of the protein N-terminus were specified as variable modifications. The instrument selected was the Orbitrap. The main search peptide tolerance and MS/MS tolerance were set to an initial mass tolerance of 4.5 ppm and 20 ppm, respectively. The minimal peptide length was set to seven amino acids, and a maximum of two mis-cleavages was allowed. Peptide identifications and protein identifications were accepted an FDR <1.0%. Label-free quantification was processed by the LFQ option.

### 3.3. RNA Extraction and RNA-Seq

The genomic RNAs were isolated from the cells using the TRIzol RNA extraction reagent (Ambion, Austin, TX, USA), following the manufacturer’s instructions. The sequencing libraries were constructed using the TruSeq RNA Sample Preparation Kit Guide (Illumina, San Diego, CA, USA) and sequencing was carried out using the Illumination Genome Analyzer IIx. Following this, the high-quality reads were mapped on hg19 in the UCSC genome browser using. The reads mapped to splice variants of one gene were summed, and the expression level of transcripts was estimated using our customized Python code, which calculates the reads per kilobase per million reads (RPKM).

### 3.4. Bioinformatic Analysis

KEGG pathway and GO enrichment analysis of the differentially expressed proteins were conducted according to the information from the KEGG Pathway and GO databases, respectively, using the following formula:

(1) $$p=1-\sum_{i=0}^{m-1}\frac{\left(\begin{array}{c} M\\ i \end{array}\right)\left(\begin{array}{c} N-M\\ n-i \end{array}\right)}{\left(\begin{array}{c} N\\ n \end{array}\right)}$$

*N* is the number of all identified proteins that can be connected with GO or KEGG Pathway analysis information. *n* is the number of differential proteins in *N*. *M* is the number of proteins that can be connected with a certain GO term or pathway. *m* is the number of differential proteins with a certain GO term or KEGG pathway. If the *p* value is below 0.05, we regard this GO term or pathway as a significant enrichment of differential proteins.

NetworkAnalyst was used to construct and visualize the differentially expressed proteins between testis and epididymis, the corresponding Protein–Protein Interaction (PPI) network was extracted, and GO was used for biological function analysis in the top module.

### 3.5. Development of the MRM-Based Targeted Proteomics Method

Amino acid sequences of 34 beta-defensin were retrieved from the UniProt database and were subjected to analysis by the PeptideCutter software. The list was shown in Table S5 in the Supplementary Materials. The protein-specific, unique tryptic peptide sequences were synthesized, and 1 pmol of each crude peptide was mixed with the 15 internal peptides. MRM translations for the selected beta-defensin peptide were performed by the Skyline software according to the former spectra library.

### 3.6. Absolute Quantification of Human β-Defensins by MRM Approach

Nine SIS according to five beta-defensins were synthesized (Bankpeptide, Ltd., China). The list of transitions is shown in Table S6 in the Supplementary Materials. The amount of SIS peptide of four to five orders of magnitude was added to digested serum proteins and tested triply to construct a standard curve. All the MRM analysis was performed on a 6500 QRTAP hybrid triple quadrupole/linear ion trap mass spectrometry (AB Sciex, CA) interfaced with UPLC systems (Eksigent, CA) using a 15 cm-long column (75 um id × 150, C18). Solvent A was 0.1% formic acid in the water, solvent B was acetonitrile containing 0.1% formic acid. The flow rate was 300 nL/min, and a 45 min water/acetonitrile gradient combined with a continuous increase of solvent B from 1% to 35% was applied. The spray voltage was 2800 V, the ion source temperature was 110 °C and the dwell time was 10 ms. All the MRM data were processed using Skyline software. The endogenous/SIS peptide ratios were calculated by the software.

## 4. Conclusions

In this study, we applied deep proteomics and transcriptomics analysis on the human testis and epididymis. In the identified 11,427 protein groups, we found 1041 testis-specific proteins and 403 epididymis-specified proteins. Further multi-omics analysis revealed the functional differences between testis and epididymis that were related to spermatogenesis, male gamete generation, as well as reproduction. Interestingly, the SARs-COVID-2 receptor ACE2 was found to express with organ specificity, which was very important information for future studies and research. More importantly, the in-depth proteomics data provided proteomic evidence for 110 MPs (neXtProt 2017.2). Furthermore, five beta-defensins were verified and quantified with the MRM technique. Altogether, this study can help researchers understand the physiological functions of testis and epididymis more deeply.

## Figures and Tables

**Table 1:** The quantitative results of five beta-defensin proteins in the testis tissue.

Accession Number	Gene Name	Testis 3 (ng/500 μg)	Testis 4 (ng/500 μg)
Q8N690	DEFB119	9.47	7.70
Q8N688	DEFB123	135.52	247.70
Q96PH6	DEFB118	-	1.76
Q8NG35	DEFB105B	2.27	1.65
Q8N104	DEFB106A	0.62	1.98

## Data Availability

The data are available by the proteomeXchange consortium repository at the site: https://www.iprox.org and the proteomeXchange ID is PXD026440.

## Supplementary Materials

The following are available online. Figure S1: RNA-Seq profiling and analysis of missing proteins, Table S1: Quantitative proteome, Table S2: Quantitative of DEGs, Table S3: HPA database, Table S4: MPs in proteomics profiling, Table S5: Peptides list of the 34 HBD, Table S6: Transition list of MRM method.

## Sample Availability

Samples of the compounds are not available from the authors.
