# Supplementary material for: A Multi-Omics Study of Human Testis and Epididymis
Source: Molecules. 2021 Jun 2;26(11):3345. doi: 10.3390/molecules26113345 (PMC8199593; doi:10.3390/molecules26113345)
Supplement: Supplementary file 1 [file molecules-26-03345-s001.zip › Supplementary-20210417.pdf]

Supplementary Materials

A Multi-omics Study of Human Testis and Epididymis

Weimin Zheng [1], Yang Zhang [2], Chuanyu Sun [3], Shengyang Ge [3], Yifan Tan [3], Huali Shen [2*], and Pengyuan Yang [1,2*]

[1] Department of Chemistry, Fudan University, Shanghai 200433, P. R. China; wmzheng6@163.com; ppyang@fudan.edu.cn
[2] Department of Systems Biology for Medicine and Institutes of Biomedical Sciences, Shanghai Medical College, Fudan University, Shanghai 200032, P. R. China; zhangyang@fudan.edu.cn; shenhuali@fudan.edu.cn; ppyang@fudan.edu.cn
[3] Department of Urology, Huashan Hospital, Fudan University, Shanghai, 200040, China; tyf930501@163.com; 12301010002@fudan.edu.cn; zhugexianglong@163.com
* Correspondence: Huali Shen; shenhuali@fudan.edu.cn and Pengyuan Yang; E-mail: pyyang@fudan.edu.cn.; Tel.: 086-021-65642009;

Table of contents

Abbreviation Index		3
Figure S1	RNA-Seq profiling and analysis of missing proteins	4
Table S1	Quantitative proteome	
Table S2	Quantitative of DEGs	
Table S3	HPA database	
Table S4	MPs in proteomic profiling	
Table S5	Peptides list of the 34 HBD	
Table S6	Transition list of MRM method	

Abbreviation Index

C-HPP	The chromosome-centric Human Proteome Project
DTT	Dithiothreitol
FASP	Filter aided sample preparation
GO	Gene ontology
HCD	High energy collisional dissociation
HPLC	High Performance Liquid chromatography
LC	Liquid chromatography
MPs	Missing proteins
MRM	Multiple reaction monitoring
MS	Mass spectrometry
MS/MS	Tandem mass spectrometry
PTMs	Post-translational modifications
RNA-Seq	RNA Sequencing
ROCs	Receiver operation characteristic curves
SDS	Sodium dodecyl sulfate
SIL	Stable isotopically labeled
SIS	Stable isotope-labeled standard
XIC	Extracted ion current

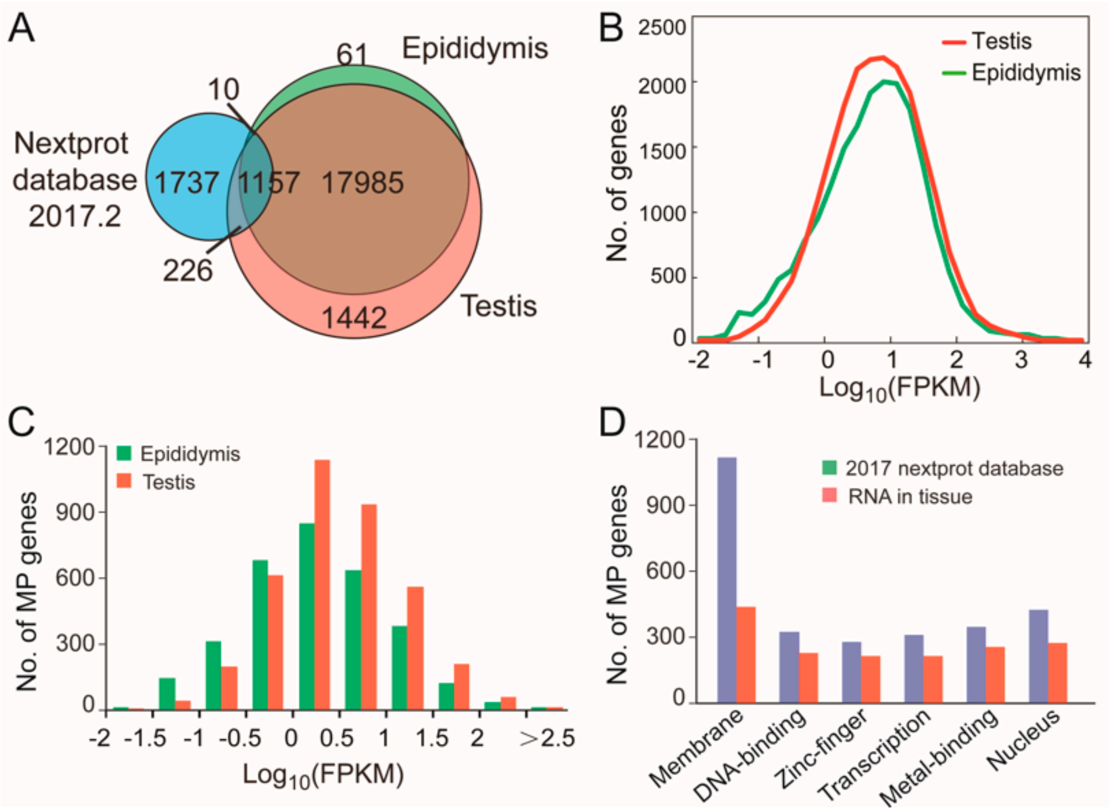

Figure S1. RNA-Seq profiling and analysis of missing proteins. (A) Venn diagram of testis and epididymis transcripts; (B) Comparison of RNA abundance distribution among the two tissues; (C) Comparison of missing proteins at the RNA expression level; (D) Enrichment compassion of missing proteins in our study and Nexprot.
